# A Treatise upon Ulcers of the Legs; in Which Former Methods of Treatment Are Candidly Examined, and Compared with One More Rational and Safe: Proving That a Perfect Cure May Generally Be Effected More Certainly, without Rest and Confinement, Than by the Strict Regimen in Common Use. With an Introduction on the Process of Ulceration, and the Origin of Pus Laudabile. To Which Are Added, Hints on a Successful Method of Treating Some Scrophulous Tumours; and the Mammary Abscess and Sore Nipples of Lying-In Women

**Published:** 1783

**Authors:** 


V. *A Treatife upon ulcers of the legs; in which former methods of treatment are candidly examined, and compared with one more rational and fafe; proving that a perfect cure may generally be effected more certainly, without reft and confinement, than by the ftrict regimen in common ufe. With an introduction on the procefs of ulceration, and*

*and the origin of pus laudabile. To which are added, Hints on a ſucceſsful method of treating ſome ſcrophulous tumours; and the mammary abſceſs and ſore nipples of lying-in women. By* Michael Underwood, *ſurgeon to the Britiſh Lying-in hoſpital.* 8vo. Matthews, *London,* 1783. 158 pages. 3 s. *in boards.*

THE author of this work conſiders pus as a ſecretion *ſui generis,* from the ruptured veſſels of a cavity or ulcerated ſurface, conſequent on a certain degree of inflammation. He ſuppoſes that in the generality of caſes ulcers of the legs are mere local complaints, not connected with any particular diſeaſe of the ſyſtem, and of courſe that leſs is to be hoped for from medicines than is commonly imagined. His plan of cure conſiſts in recommending exerciſe, a free generous diet, and ſtrong ſtimulating ointments to the ſore, aſſiſted by a tight roller, made of Welch flannel, three inches wide, and carried from the end of the foot to the knee.

In the cure of an ulcer, he obſerves, the firſt object of the ſurgeon muſt be to bring it to diſcharge a laudable pus, and this, he aſſerts, the moſt inveterate ulcers on the legs may be brought to afford, as freely as ſores ſeated any where where elfe. He recommends the *mer. corrof. ruber.* finely levigated, as one of the beft applications for this purpofe. " It may be faid of " this"—obferves Mr. Underwood—" as Hip- " pocrates fays of frictions, that it foftens the " hard, ftrengthens the relaxed fibres, deftroys " the unfound, ftimulates the growing flefh; " promotes, or diminifhes difcharge, and keeps " open, or heals up the ulcer, juft as you would " direct it." But for fome of thefe purpofes, he adds, " it muft be ufed in great quantity, " and inftead of being lightly fprinkled over an " ill-conditioned furface, the ulcer muft be " filled with it, the furgeon taking up a large " pinch of it, and boldly plugging up the fore." He acknowledges that this is a homely mode of dreffing a wound, but experience of its efficacy has reconciled him to it. This liberal application of precipitate to ulcers is recommended by Wifeman, and our author is perfuaded that both Wifeman and Turner owed much of their fuccefs to it.

Mr. Underwood fpeaks of a fpecies of ulcer which is ufually very fmall, and particularly affects the parts about, and even below the ancle. It is exquifitely painful, and for fome time he found it difficult of cure without refting the leg, till till at length by carrying the roller ſeveral times over the ancle and foot, ſo as to leave no part but juſt the point of the heel uncovered, he was enabled to make a tolerable compreſſion below the ulcer itſelf. Caſes of this ſort, he obſerves, "are often attended with conſiderable puffineſs "and a tetterous appearance of the ſurrounding "ſkin, accompanied with a thin and acrid diſ-"charge, which render the parts additionally "tender; whilſt the little ulcer is almoſt per-"fectly dry, and cannot eaſily be brought to "ſuppuration, till the complaint of the ſkin is "removed." This, we are told, is moſt ſpeedily effected by drying applications, ſuch as bole and powdered alum, *ung. rubr. deſiccativ.* or in more obſtinate caſes, a ſolution of ſugar of lead and white vitriol, with an ounce or two of camphorated ſpirit in a pint of water. If the ſore does not ſoon change its complexion, on the diſappearance of the affection of the ſkin, we are adviſed to fill the ulcer with precipitate, diſſolved lunar cauſtic, or any ſimilar eſcharotic, and when the ſlough is come out to repeat it. Here, however, our author offers a caution relative to the uſe of theſe eſcharotics, which he obſerves are to be had recourſe to only where active digeftives, aided by proper bandage and exercife, prove ineffectual.

In ulcers of long ftanding, it fometimes happens, that befides a large fore, the leg is exceedingly fwelled with hard tumors, or lumps, which will not always be diffolved by the difcharge from the ulcer. Here, we are told, the application of a large piece of oiled filk will produce the happieft effects, and with fafety difperfe the indurations. It likewife fometimes happens, that the other leg will be equally fwollen, and exceedingly hard, though without ulceration. In this cafe alfo, whilft a copious difcharge is kept up from the fore, the tumid leg is directed to be rolled, and covered with oiled filk, which, by exercife, will, without the affiftance of medicine, bring down the hardnefs and fwelling, by the time the ulcer on the other leg is healed.

Wherever ulcers are connected with difeafe of the fyftem, our author allows, that the affiftance of medicine will be required. All that he means to infift on is, that this is not generally the cafe. In one kind of ulcer, ufually of long ftanding, and which he thinks has been improperly termed fcorbutic, he has found the bark very efficacious when given in large dofes. This fort of ulcer, he obferves, occurs chiefly in poor people, who have have either injured their conſtitution by hard drinking, or, on the contrary, have been in want of almoſt all the neceſſaries of life. In caſes of obſtructed perſpiration and cutaneous eruption, he has experienced good effects from a ſtrong decoction of the woods, and in eryſipelatous ulcers, eſpecially where a great part of the limb is infeſted with a ſcalding diſcharge, he has recommended the internal uſe of lime water with advantage.

In the healing of ulcers, eſpecially thoſe of long ſtanding, Mr. Underwood adviſes the ſurgeon to proceed ſlowly and cautiouſly, avoiding the too early uſe of drying applications, and gradually weakening the digeſtive. It may be laid down as a general maxim, he obſerves, that the ſore ſhould rather be ſuffered, than invited to ſkin over. When the ulcer is healed, he recommends temperance, a continuance of the bandage for ſome time, and purgatives occaſionally.

The good effects of precipitate and warm digeſtive ointments, aſſiſted by a generous diet and exerciſe, in ulcers of the legs, induced our author to extend the trial of them to thoſe glandular tumours about the neck, which have generally, though perhaps not on ſufficient grounds, been confidered as fcrophulous. His experiments in this way, he affures us, have been attended with fuccefs. If the fwellings are at all difpofed to come forward, but are not broke, or have only a fmall orifice, he always haftens the maturation, and the diffolution of the fkin as far as it is difeafed, by epithems made of honey, flour, and yolk of egg. He is very little concerned to what extent the fore fhall run, as he knows he fhall have much diftempered gland to deftroy underneath, and that if the latter be not effectually done, the fore will either not heal entirely, or will foon break out again. As he fuppofes the difeafe to be local, he prefcribes no mercurial or other medicine internally, unlefs the patient is unhealthy in other refpects. The frequent purges, which are fo generally recommended, he thinks, ferve only to reduce the vis vitæ which, in thefe cafes, is already too languid.

To obtain the advantages Mr. Underwood has experienced from the ufe of precipitate in thefe cafes, the ulcers—he tells us—are to be filled with it, and if a flough is formed by it (which will not always be the cafe) the fuppurative epithem is recommended as the beft dreffing till the flough is thrown off, when the precipitate cipitate is to be immediately repeated. It frequently happens, he adds, that fcrophulous ulcers, cured by this method, leave only a feam, and a little rednefs to be obferved afterwards, without any proper fcar on the part.

On this fubject the editor of the Journal begs leave to obferve, that he himfelf, about three years ago, having had a tumour of this kind in his neck, which came flowly to fuppuration, and having long been perfuaded that complaints of this fort are in general local, and unconnected with any difeafe of the fyftem, he treated it accordingly. At the end of a few months, when the tumour had acquired the fize of a pigeon's egg, and the fkin was become thin, foft, and pointed, it burft of itfelf, and the opening was a little enlarged with a lancet fo as to afford a free outlet to the matter. As there ftill remained much indurated gland, one of his furgical friends advifed him to deftroy it with a cauftic, but he chofe rather to truft to time and to milder applications. At firft the *Linimentum Arcæi* fpread on lint was applied to the wound, but as this conftantly irritated the fore, and made it inflamed and painful, it was after feveral trials laid afide, and in its ftead recourfe was had to wax and oil; by which fimple application, without out any aſſiſtance from medicine, the wound continued to diſcharge for ſeveral months, without the leaſt pain or uneaſineſs, and at length, at the end of about a year, was completely healed, the induration of the gland being then totally removed. During the whole of this period, his health, which has always been good, was unaffected, and he continued to live as uſual, taking a great deal of exerciſe and eating freely of animal food. A redneſs of the ſkin at the part and a ſlight ſcar ſtill remain.

From this and ſome other ſimilar caſes which have come under his obſervation, and in which he has recommended a ſimilar practice, he is convinced, that as little or nothing is to be expected from medicine, ſo likewiſe that the leſs the ſurgeon interferes with them the better, as they generally ſucceed beſt when left to nature, aſſiſted by the mildeſt applications.

We now return to Mr. Underwood's work, towards the concluſion of which we meet with ſome obſervations on ſore nipples and the mammary abſceſs. In the firſt of theſe he recommends the aſtringent ſolution mentioned p. 257, and obſerves, that he has found ſome advantage in theſe caſes, and in drawing out a bad nipple, by covering it with a large nutmeg, hollowed out, out, with the edges left flat. In the treatment of the mammary abſceſs which, he obſerves, is often the conſequence of neglected inflammation of the nipples, he very properly cautions us againſt the old, and now, we hope, generally exploded practice, of making a large or indeed any opening with an inſtrument. All that is commonly neceſſary, he remarks, is to cover the part with a ſoft bread and milk poultice; to keep it well ſupported by an eaſy bandage; and carefully to preſs out the matter, and renew the poultice twice or three times a day. He has met with ſome inſtances, however, where ſmall punctures with a lancet have been uſeful. Such were caſes, where the whole breaſt was very hard, and the habit not diſpoſed to form matter ſo plentifully as the indurated ſtate of the part ſeemed to require. In ſome alarming caſes, where the breaſt had entirely loſt its natural appearance, and was become hard in every part, flattened, and the nipple almoſt obliterated, he has ſeen nothing ſo uſeful as mild poultices and a cautious uſe of the Ung. Merc. fort. with occaſional V. S. and the uſe of laxatives.

VI. *Phar-*

